# Body Composition and Cardiorespiratory Fitness vs. 1‐MET Value in Older Adults: A Factor Analytic Study

**DOI:** 10.1155/jare/2866677

**Published:** 2026-08-18

**Authors:** Gabriela Venturini, Paulo Farinatti, Carlos A. A. Ribeiro, Felipe A. Cunha, Nádia S. L. Silva

**Affiliations:** ^1^ Federal Center for Technological Education of Minas Gerais (CEFET-MG), Leopoldina, State of Minas Gerais, Brazil; ^2^ Graduate Program in Physical Exercise and Sports Sciences, University of Rio de Janeiro State, Rio de Janeiro, State of Rio de Janeiro, Brazil

**Keywords:** aging, factor analysis, metabolism, principal component analysis, VO_2_

## Abstract

**Background:**

The metabolic equivalent (1‐MET) represents the oxygen consumption at rest, reflecting the body’s minimal energy needs. Determinants of 1‐MET among older adults are yet to be defined. This cross‐sectional study examined the association between 1‐MET, body size and composition, and cardiorespiratory fitness in older adults using principal component analysis (PCA).

**Methods:**

Fifty‐eight participants aged 60 and above (12 males, 75.4 ± 6.5 years) were assessed for 1‐MET and peak oxygen consumption (VO_2peak_) via indirect calorimetry, and body composition was measured by dual‐energy X‐ray absorptiometry.

**Results:**

The average 1‐MET value (2.3 mL·kg^−1^·min^−1^) was significantly lower than the reference 3.5 mL kg^−1^·min^−1^ (*p* < 0.001). PCA identified two factors accounting for 79.3% of the variance. Factor 1 (51.2% variance) grouped body mass index (BMI), fat mass, and body surface area. Factor 2 (28.1% variance) linked VO_2peak_ and 1‐MET. Results showed that 1‐MET was more strongly associated with cardiorespiratory fitness than body size or composition.

**Conclusions:**

Cardiorespiratory fitness seems to be a determinant of the resting metabolic rate in older adults, and lower 1‐MET values would be expected in individuals with poorer vs. higher VO_2peak_.

## 1. Introduction

The metabolic equivalent (1‐MET) is a physiological concept that describes the energy cost at rest, usually expressed as a relative oxygen consumption (VO_2_) of 3.5 mL kg^−1^·min^−1^ [1]. The intensity of physical activities is often expressed as multiples of the 1‐MET, the ratio between the working and resting metabolic rate. Although useful in epidemiological and clinical sets, the 1‐MET reference value has been criticized due to considerable differences between specific populations [2]. Factors such as body mass, body surface, fat‐free mass, hormonal levels, and cardiorespiratory fitness influence VO_2_ at rest and are affected by aging [3].

Studies investigating the 1‐MET among older adults reported that it tends to be lower than the widely accepted reference value [1, 2]. Changes in body composition, particularly body mass and fat‐free mass, have been acknowledged as determinants of decreased resting metabolism throughout aging [4]. On the other hand, there is evidence that factors other than changes in body size or composition concur with age‐related unfavorable modifications in resting metabolism [5]. Examples are lower anabolic hormonal pulsatility [6] or decreases in the energy expenditure of organs and physiological systems [7].

Although the determinants of resting metabolic rate are well known, additional research is warranted to identify the effects of aging on their relative influence. Most trials investigating the variation of 1‐MET in older adults focused on the role of body size and composition [4] and produced mixed results—for instance, there are doubts concerning the contribution of overall body weight or fat vs*.* fat‐free mass. On the other hand, there is a consensus that physical activity entails body movement that increases overall energy expenditure and cardiorespiratory capacity [4]. Accordingly, higher metabolism at rest has been reported in individuals with moderate to high vs. low cardiorespiratory fitness [8]. Regular endurance training seems to accelerate the metabolism at the cellular level [7]. Albeit the rationale that indicates a theoretical relationship between cardiorespiratory fitness and resting metabolism, trials investigating its specific contribution to the 1‐MET value in older adults are scarce and produced mixed findings [7, 8]. A better understanding of the relationships between body composition, cardiorespiratory fitness, and 1‐MET with aging would be useful for establishing expectations regarding the effects of health‐related interventions on the metabolism of older people.

The principal component analysis (PCA) is a strategy that allows reducing many variables in a dataset, improving the visualization of the major dimensions underlying their common variation. The dimensionality of several interrelated variables is simplified while retaining as much of the variation in the dataset. It would be useful to analyze the relationship of the 1‐MET value vs. a large set of variables theoretically associated with age‐related changes in the resting metabolic rate through PCA. Variables showing closer communality vs. resting VO_2_ (e.g., grouped in a common factor) could be interpreted as stronger determinants of 1‐MET. We could not find prior research adopting this approach to investigate variables influencing the 1‐MET in older adults.

Given this gap in the literature and accepting the premise that reductions in the 1‐MET value with aging would result from changes in body size, body composition, and cardiorespiratory fitness, this study investigated through PCA the relationship of resting VO_2_ (or 1‐MET) and markers of those variables in subjects aged ≥ 60 years. We hypothesized that body size and body composition alone would not be the best predictors of the resting metabolic rate and therefore the 1‐MET value in older males and females.

## 2. Materials and Methods

### 2.1. Sample

Sample calculation (G∗Power 3.1.5, University of Kiel, Germany) was performed using a bivariate normal model and exact distribution with a correlation *ρ* H1 of 0.5, power of 0.80, and an alpha level of 0.05. A minimum of 29 participants was found necessary. We measured 58 subjects (12 males) aged 62–91 years (75.4 ± 6.5 years; 26.7 ± 4.4 Kg/m^2^) who dwelled in the community. Volunteers were recruited by convenience from a single university‐affiliated center and volunteered to participate in the study. Therefore, the sample may not fully represent the broader older adult population, particularly individuals with different socioeconomic, clinical, or functional characteristics.

Eligible individuals were community‐dwelling older adults aged ≥ 60 years, nonsmokers, and clinically stable at the time of the assessments. Exclusion criteria included known heart or pulmonary disease, uncontrolled metabolic disorders (e.g., uncontrolled diabetes or thyroid disease), acute illness, cognitive impairment precluding understanding of study procedures, and locomotor limitations preventing safe exercise performance. Participants using medications with potential acute effects on resting metabolism or exercise responses underwent medical screening, and all volunteers were instructed to maintain their usual pharmacological treatment throughout the study period. Health information, including medical history and medication use, was obtained from medical records with the agreement of both participants and their physicians.

All volunteers provided written informed consent prior to participation. The study was approved by the University Hospital Ethical Committee (CAAE: 94336418.2.0000.5259, date of approval August 14, 2018).

### 2.2. Study Design

Data assessment included three visits interspersed over 48–72 h. On the first visit, subjects signed informed consent and underwent clinical examinations. Subsequently, anthropometric and body fractioning assessments were performed. On the second visit, the VO_2_ at rest was quantified following strict recommendations [9] to determine individual 1‐MET values. On the third visit, maximal cardiopulmonary exercise testing (CPET) was applied to determine the VO_2_ at maximum effort (VO_2peak_).

### 2.3. Anthropometric and Body Fractioning Assessments

Body mass and height were respectively assessed by a digital balance scale (Filizola, São Paulo, SP, Brazil) and a stadiometer graded in millimeters (Sanny, São Bernardo do Campo, SP, Brazil). The body mass index (BMI) (Kg/m^2^) and body surface area (m^2^) were calculated. Fractions of body composition were determined through dual‐energy X‐ray absorptiometry (DXA) (Lunar iDXA, GE Healthcare, Chalfont St. Giles, United Kingdom). The following variables were used: body mass (kg), fat mass (g), and fat‐free mass (g). The appendicular skeletal muscle mass (ASM) was calculated as the sum of muscle mass of legs and arms.

### 2.4. VO_2_ at Rest

Resting oxygen uptake (VO_2_) was determined in the laboratory following strict recommendations [9]. In brief, this consisted of abstention from physical exercise, alcohol, soft drinks, and caffeine in the 24 h preceding the assessments, fasting for at least 3 h, and performing minimum effort when going to the laboratory. Participants laid supine in a controlled environment (21°C–23 °C, 50%–70% air humidity) for an acclimation period of 10 min, after which VO_2_ was determined for 30 min.

Assessments took place at the same time (7–11 a.m.) to negate circadian variations in the metabolism. Minute ventilation and pulmonary gas exchange were determined breath‐by‐breath using a face mask connected to an Ultima CardiO_2_ metabolic cart (Medical Graphics, Saint Louis, MO, USA). The gas analyzers were calibrated before each test according to the manufacturer’s instructions using a certified standard mixture of oxygen (17.01%) and carbon dioxide (5.00%), balanced with nitrogen (AGA, Rio de Janeiro, RJ, Brazil). The accuracy of the flows and volumes estimated by the pneumotachometer were verified using a 3‐L graduated syringe (Hans Rudolph, Kansas, MO, USA). The analysis considered the average of the last 5 min of VO_2_ data (coefficient of variation ≤ 10%) since it has already been demonstrated that this period presents high test–retest reliability [2].

### 2.5. CPET

The VO_2peak_ was determined using a ramp‐incremented protocol until each participant’s tolerance on an electronically braked cycle ergometer (Inbrasport, Porto Alegre, RS, Brazil). Subjects were instructed to avoid any physical effort in the 24 h, fast for 3 h, and not consume alcoholic or caffeinated beverages in the previous 8 h to the test. Final work output and work rate increments were calculated using the ACSM’s metabolic equation for cycling [10] based on a target time to exhaustion of 10 min.

The work rate for the 3‐min warm‐up period was 0 W, and the initial work rate was 30 W. Cycling cadence was maintained at 55–65 rpm. The CPET was performed until maximal tolerated effort, as determined by volitional exhaustion reflected by a score of 9 on the Borg CR10 scale or inability to maintain a cycling cadence ≥ 55 rpm despite verbal encouragement.

The heart rate was continuously measured using an electrocardiogram (Welch Allyn, Skaneateles Falls, New York, USA). Gas exchange data were acquired every three complete respiratory cycles (∼10 s) via the Ultima CardiO2 metabolic cart (Medical Graphics, Saint Louis, USA), and the VO_2peak_ was defined as the highest VO_2_ value attained during a 30‐s interval of the CPET. A facemask (Hans Rudolph, Kansas, MO, USA) covering the mouth and nose was attached to a bidirectional digital flow valve and fastened by Velcro straps. The gas analyzer and pneumotachograph were calibrated according to the manufacturer’s instructions. The ambient temperature ranged from 21°C to 23°C and the relative humidity from 50% to 70%.

### 2.6. Statistical Analysis

Data normality was assessed by the Kolmogorov–Smirnov test and results are presented as mean ± standard deviation. Student’s *t*‐tests for independent samples were applied to compare the sample characteristics between males and females. Potential differences between the actual 1‐MET vs. reference value (3.5 mL·kg^−1^·min^−1^) were verified by the one‐sample Student’s *t*‐test. Pearson correlations between 1‐MET and markers of body size (BMI and surface area), body composition, and cardiorespiratory fitness were calculated for the whole sample and each sex. In addition, 95% confidence intervals for the correlation coefficients were calculated using Fisher’s z transformation.

Prior to the PCA, the dataset was screened for missing values, influential outliers, and violations of linearity assumptions. No missing data or influential outliers were identified, and the relationships among variables were considered sufficiently linear to support the use of correlation‐based dimension reduction techniques. Variables showing significant associations were included in the PCA after checking the sampling adequacy for each variable using the Kaiser–Meyer–Olkin test (KMO). The KMO reflects the proportion of variance among variables that might show common variance. Higher KMO values reflect a greater proportion of common variance, indicating that the data would be more suited to factor analysis. KMO values lower than 0.6 indicate inadequate sampling and that remedial action should be taken [11]. In addition, Bartlett’s sphericity test provided information about whether the correlations in the data were strong enough to use a dimension‐reduction technique. If the variables are weakly correlated, the *p* value is not significant, and PCA would not be possible.

The varimax rotation was applied to extract factors in the PCA, which is an orthogonal rotation that aids interpretability. This approach maximized the sum of the variances of the squared loadings as all coefficients would be either large or near zero, with few intermediate values. The goal was to associate each variable with at most one factor based on eigenvalues > 1.0 (Kaiser criterion). Calculations were performed using the SPSS 20.0 software (IBM, Armonk, NY, USA), and the significance level was fixed at *p* < 0.05.

## 3. Results

Table 1 summarizes the sample characteristics. Participants were predominantly female (79%), exhibited normal to excess body weight (BMI range: 19.7–38.5 kg·m^−2^), and presented poor to moderate cardiorespiratory fitness (VO_2_peak range: 5.9–27.0 mL kg^−1^·min^−1^).

**TABLE 1:** Sample characteristics (mean ± standard deviation).

	Total (*n* = 58)	Males (*n* = 12)	Females (*n* = 46)	*p* value (mean difference [95% CI])
Age (years)	75.4 ± 6.5	76.6 ± 5.2	75.1 ± 6.8	0.465 (1.57 [−2.70 to 5.83])
Body mass (kg)	65.2 ± 11.2	69.4 ± 9.9	64.1 ± 11.3	0.148 (5.25 [−1.92 to 12.41])
Height (cm)	156.3 ± 7.9	166.3 ± 6.8	153.7 ± 5.9	< 0.001 (12.57 [8.61 to 16.54])
Body mass index (kg/m^2^)	26.7 ± 4.4	25.1 ± 3.3	27.1 ± 4.6	0.154 (−2.06 [−4.92 to 0.80])
Fat mass (kg)	26.0 ± 7.7	21.4 ± 4.9	27.2 ± 7.8	0.017 (−5.88 [−10.65 to −1.11])
Fat‐free mass (kg)	39.2 ± 6.7	48.0 ± 5.7	36.9 ± 4.7	< 0.001 (11.13 [7.91 to 14.34])
ASM (kg)	16.4 ± 3.2	20.3 ± 2.9	15.3 ± 2.3	< 0.001 (5.02 [3.45 to 6.60])
Relative fat (%)	40.5 ± 7.2	31.7 ± 3.5	42.8 ± 6.1	< 0.001 (−11.15 [−14.83 to −7.47])
Body surface area (m^2^)	1.65 ± 0.15	1.77 ± 0.14	1.61 ± 0.14	0.001 (0.16 [0.06 to 0.25])
VO_2peak_ (L/min)	1.0 ± 0.3	1.3 ± 0.2	0.9 ± 0.3	< 0.001 (0.33 [0.15 to 0.50])
VO_2peak_ (mL·kg^−1^·min^−1^)	15.4 ± 4.0	18.4 ± 3.7	14.6 ± 3.7	0.003 (3.74 [1.35 to 6.13])
VO_2peak/FFM_ (mL·kg^−1^·min^−1^)	25.5 ± 6.2	26.5 ± 5.5	25.3 ± 6.4	0.546 (1.22 [‐2.81 to 5.25])
Resting heart rate (beats/min)	66 ± 10	65 ± 9	66 ± 10	0.760 (−0.98 [−7.41 to 5.44])
VO_2rest_ ‐ 1‐MET (mL·kg^−1^·min^−1^)	2.3 ± 0.5	2.5 ± 0.5	2.3 ± 0.5	0.130 (0.25 [−0.08 to 0.58])

*Note:* Values in the last column represent the mean difference (males − females) and its 95% confidence interval (95% CI) obtained from independent‐samples *t*‐tests. VO_2_rest, oxygen uptake at rest; VO_2_peak, peak oxygen uptake; VO_2_peak/FFM, peak oxygen uptake relative to fat‐free mass.

Abbreviations: 1‐MET, metabolic equivalent, ASM, appendicular skeletal muscle mass.

Compared with females, males were taller and exhibited greater body surface area, ASM, fat‐free mass, and absolute and relative VO_2_peak, whereas females had higher absolute and relative fat mass. No significant differences between sexes were observed for age, BMI, VO_2_peak normalized to fat‐free mass, resting heart rate, or 1‐MET.

The mean 1‐MET value for the whole sample was 2.3 ± 0.5 mL kg^−1^·min^−1^ (range: 1.16–3.65 mL kg^−1^·min^−1^), which was significantly lower than the conventional reference value of 3.5 mL kg^−1^·min^−1^ (*p* < 0.001). No significant sex difference was observed for 1‐MET (mean difference = 0.25 mL kg^−1^·min^−1^; 95% CI −0.08 to 0.58; *p* = 0.130). Relative to the reference value, measured 1‐MET values were approximately 1.2 mL kg^−1^·min^−1^ lower in the overall sample. The mean difference ranged from approximately −1.3 mL kg^−1^·min^−1^ in females to −1.0 mL kg^−1^·min^−1^ in males, indicating that the conventional 1‐MET value systematically overestimated resting oxygen uptake in both sexes.

**FIGURE 1:**
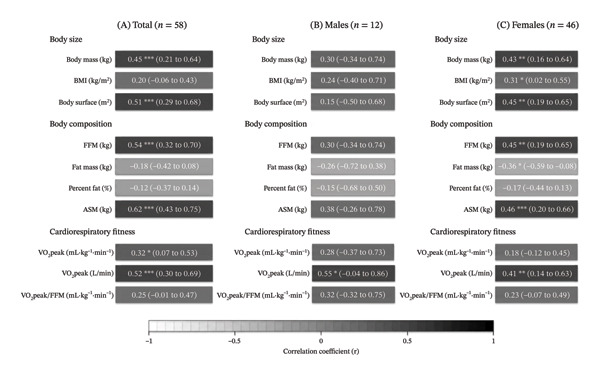
Correlations between VO_2_ at rest and variables of body size, body composition, and cardiorespiratory fitness. Panels show (A) total sample (*n* = 58), (B) males (*n* = 12), and (C) females (*n* = 46). Values are Pearson correlation coefficients (r) with corresponding 95% confidence intervals; shading reflects the magnitude and direction of correlations (−1 to +1). ^∗^ *p* < 0.05; ^∗∗^ *p* < 0.01; ^∗∗∗^ *p* < 0.001. FFM: fat‐free mass; ASM: appendicular skeletal muscle mass; VO_2_peak: peak oxygen uptake.

Figure 1 shows the correlations between VO_2_rest and variables of body size, body composition, and cardiorespiratory fitness. VO_2_rest was positively associated with body size and muscle mass and weakly or inversely associated with fat‐related variables. Positive associations were also observed with cardiorespiratory fitness. Associations with body size/composition were slightly stronger in females, whereas those with fitness were stronger in males. The heatmaps (Figure 1A–C) visually summarize these patterns.

Variables included in the PCA based on the correlation matrix were as follows: BMI, fat mass, body surface area, and absolute VO_2peak_. Albeit showing significant correlations, body mass, fat‐free mass, percent fat mass, and appendicular muscle mass were not included in the factorial model due to collinearity with the previously selected variables. The KMO of 0.692 indicated that the sample was adequate for factor analysis, while Bartlett’s sphericity test was significant (approximate chi‐square 149.66, *p* < 0.0001).

**FIGURE 2:**
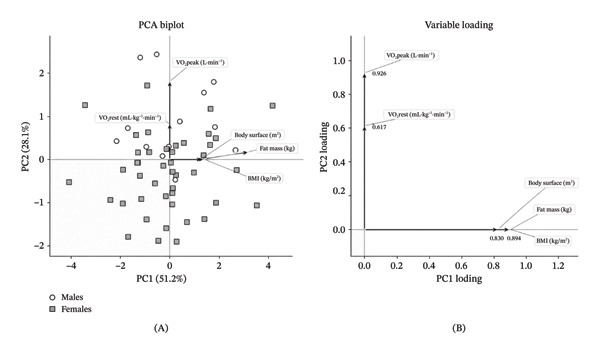
Principal component analysis (PCA) biplot and variable loadings. Panel A shows individual scores for the first two principal components, with symbols distinguishing males (○) and females (■), along with variable loadings. Panel B presents the loadings of each variable on PC1 and PC2. Markers of body size and composition (BMI, fat mass, and body surface area) load primarily on PC1, whereas cardiorespiratory variables (VO_2_peak and VO_2_rest) load on PC2. Percent variance explained: PC1 = 51.2% and PC2 = 28.1%.

Table 2 shows the results of the PCA. Components with factor loadings < 0.5 are not presented to improve visualization. Two factors with eigenvalues > 1.0 were extracted, accounting for 79.3% of the total variance. The first factor grouped markers of body size and composition, whereas the second factor grouped 1‐MET and VO_2_peak. These relationships are visually illustrated in Figure 2, where the biplot shows body size and composition variables clustering along PC1 and cardiorespiratory variables loading on PC2. In summary, the variance of VO_2_peak was more closely aligned with the variance of the 1‐MET value, suggesting that body size and composition are not the primary predictors of resting metabolic rate.

**TABLE 2:** Components, communalities, eigenvalues, and explained variance within the principal component analysis (*varimax* rotation) including the 1‐MET value and markers of body size, body composition, and cardiorespiratory fitness among older adults (*n* = 58).

	Rotated components		Communalities
	1	2	
Body mass index (kg/m^2^)	0.894		0.866
Fat mass (kg)	0.898		0.878
Body surface area (m^2^)	0.830		0.733
VO_2peak_ (L·min^−1^)		0.926	0.862
1‐MET (mL·kg^−1^·min^−1^)		0.617	0.619
Eigenvalue	2.57	1.39	
% Variance (total 79.3%)	51.2	28.1	

*Note:* VO_2peak_, peak oxygen uptake.

Abbreviation: 1‐MET, metabolic equivalent.

## 4. Discussion

This study investigated through PCA the relationship of 1‐MET value vs. variables reflecting body size, body composition, and cardiorespiratory fitness in individuals aged ≥ 60 years. The major findings were as follows: (a) in general, the 1‐MET values of older males and females were lower than the reference value of 3.5 mL kg^−1^·min^−1^ (average of 2.3 mL kg^−1^·min^−1^); (b) variables of body size (BMI and body surface), body composition, and cardiorespiratory fitness correlated with the relative (1‐MET) and absolute VO_2_ at rest; (c) fluctuations in 1‐MET among older adults had greater communality with the relative VO_2peak_ than with markers of body size and composition. These results suggest that cardiorespiratory fitness may be a major determinant of the 1‐MET value in older adults.

The reference value of 3.5 mL kg^−1^·min^−1^ for the 1‐MET has been challenged [2], and there is extensive evidence that the metabolic rate declines with aging. Even considering the great variability observed in older populations, a 3%–5% reduction per decade in the metabolism at rest has been reported [12]. The 1‐MET value presently observed concurs with previous studies with older samples. Overall, studies including healthy older samples reported 1‐MET values ranging from 0.84 to 4.41 mL kg^−1^·min^−1^ (average 2.54 mL kg^−1^·min^−1^) [13].

In studies with unhealthy individuals, the range was smaller, but the 1‐MET value was also consistently lower than the reference value—cardiorespiratory diseases (average 2.80 mL kg^−1^·min^−1^, range 2.06–3.88 mL kg^−1^·min^−1^) [14, 15]; cancer (average 2.87 mL kg^−1^·min^−1^, range 2.25–3.61 mL kg^−1^·min^−1^) [16, 17]; chronic obstructive pulmonary disease (average 2.96 mL kg^−1^·min^−1^, range 2.41–3.60 mL kg^−1^·min^−1^) [18, 19]; hospitalized elderly (average 2.75 mL kg^−1^·min^−1^, range 2.15–3.58 mL kg^−1^·min^−1^) [20–22]; chronic kidney failure (average 2.54 mL kg^−1^·min^−1^, range 1.75–3.13 mL kg^−1^·min^−1^) [23, 24]; obese elderly (average 2.85 mL kg^−1^·min^−1^, range 2.64–3.08 mL kg^−1^·min^−1^) [25, 26]; frail individuals (average 2.53 mL kg^−1^·min^−1^, range 2.10–2.87 mL kg^−1^·min^−1^) [12, 27]; Alzheimer (average 1.16 mL kg^−1^·min^−1^) [28]; stroke (average 2.86 mL kg^−1^·min^−1^, range 2.67–3.05 mL kg^−1^·min^−1^) [29, 30]; femur fracture (average 3.61 mL kg^−1^·min^−1^) [31]; individuals with testosterone, estrogen, or GH deficits (average 2.66 mL kg^−1^·min^−1^, range 2.46–2.76 mL kg^−1^·min^−1^) [32, 33], or with one or more noncommunicable diseases (average 2.70 mL kg^−1^·min^−1^, range 2.69–2.72 mL kg^−1^·min^−1^) [34].

Our sample was predominantly composed of women (79%) recruited from a single geographic region. Although no significant sex differences were observed for the 1‐MET value, males and females differed in body size, body composition, and cardiorespiratory fitness, factors known to influence metabolic responses and energy expenditure [5, 35]. Therefore, sex‐related physiological differences may have influenced the observed correlation structure and, consequently, the PCA solution. Because the small number of males precluded reliable sex‐specific PCA solutions, the factor structure should be interpreted as reflecting the overall sample, which was predominantly female, rather than as evidence of identical metabolic relationships in both sexes. To mitigate this possibility, variables were selected for the PCA based on significant correlations observed in both the overall sample and sex‐stratified analyses. Moreover, correlations with absolute VO_2_ at rest were calculated to minimize the potential influence of body dimensions and muscle mass on the observed relationships. Finally, collinearity levels were assessed to prevent overemphasis on one variable at the expense of others. Notably, body mass and BMI exhibited clear interdependence, as did fat mass and fat‐free mass, as well as absolute and relative VO_2_peak. Given the predominance of women and the recruitment of participants from a single geographic area, the present findings should be interpreted within the context of this specific sample and may not be directly generalizable to broader and more diverse older populations.

Prior research investigated the potential associations of body composition with resting metabolism in older adults. Decreases in body mass, BMI, or fat‐free mass (with a consequent increase in relative fat mass) are often suggested as determinants of the resting metabolic rate and major predictors of the 1‐MET value [4]. In general, the literature attributes age‐related changes in the metabolism to losses in skeletal muscle (sarcopenia) [3]. However, there is evidence that changes in the resting metabolism are not entirely due to body composition [2, 5, 7]. Kwo et al. [5] showed an increase in body fat of 1.7 kg/decade, concomitant to a linear decrease in the 1‐MET during the same period. Some authors claimed that the fat‐free mass metabolism is not homogeneous and may vary according to the energy expenditure of specific organs and tissues and their relative proportion [35]. In short, the influence of aging on the metabolism involves a complex interaction of systems beyond the role of variables related to body size and composition—decreases in the resting metabolic rate seem to result not only from changes in fat‐free mass but also due to slowed organ metabolic activity [7].

More recently, it has been suggested that the frailty status could also modulate the energy requirements of the elderly—at least one population‐based study demonstrated that frail and prefrail older adults presented lower resting metabolic rates than nonfrail counterparts of similar age [12]. Besides, it is well accepted that the decline in the resting metabolic rate may be attenuated in individuals who maintain a high exercise volume or energy intake throughout their life span [3]. Also, reductions in fat‐free mass may be partially related to decrements in VO_2peak_ and resting metabolic rate [4, 21]. These premises support that physical fitness levels relate to resting metabolism.

Prior studies suggested a 5%–20% higher metabolic rate in highly fit vs. sedentary subjects [36]. In addition, associations with cardiorespiratory fitness have been demonstrated in different populations [36, 37]. The present findings expand the current knowledge by investigating the association of body size/composition and cardiorespiratory fitness with the 1‐MET value in older individuals exhibiting a wide range of nutritional status and VO_2peak_. Our results are unique given that no previous studies have explored these associations using a dimension‐reduction technique such as PCA. Two factors explained 79.3% of the total variance in the input variables, indicating an effective dimensionality reduction [11]. Variables related to body size and composition accounted for approximately 51% of the variance, while VO_2peak_ and 1‐MET formed a distinct factor, explaining an additional 28% of the variance.

The consistent associations between body size and composition vs*.* 1‐MET ratify current evidence [4]. However, the predictive value of these variables is mixed and has been challenged [1, 38]. The 1‐MET throughout aging reflects the energy demand of several body systems [35] and should not be attributed to a specific variable [5]. Our data are suggestive that variations in the maximal cardiorespiratory capacity are closely related to fluctuations in the resting metabolic rate among older adults—hence, individuals with greater VO_2peak_ would be expected to exhibit higher 1‐MET values. This agrees with studies questioning the role of body weight or fat‐free mass as major determinants of the 1‐MET and with trials proposing that age‐related changes in 1‐MET and VO_2peak_ would be proportional [36, 37]. The hypothesis that enhanced cardiorespiratory capacity reflects physiological adaptations leading to increased energy expenditure at rest was thus supported [3, 39].

This study has strengths and limitations. Our findings advance the current knowledge by originally demonstrating through factor analysis that cardiorespiratory fitness may be a key determinant of the resting metabolic rate in older individuals. This possibility has implications not only for the metabolism assessment but also for exercise interventions to improve the resting metabolic rate in this group.

The major limitation of this study was the relatively small sample, which may restrict the generalizability of the PCA findings. Nevertheless, sampling adequacy was supported by the KMO test, and the sample size met the standard recommendation of 5–20 observations per variable [40]. In addition, the predominance of women in the sample (79%), single‐center recruitment, and volunteer‐based participation may have introduced selection bias and further limited the external validity of the findings. Since the sample size was originally estimated for correlation analyses rather than multivariate factor extraction, the PCA results should be interpreted as exploratory. Furthermore, the combination of a relatively small sample (*n* = 58) and multiple interrelated variables may have increased susceptibility to overfitting. The lack of control for factors potentially influencing the 1‐MET value, such as diet and physical activity levels, should also be acknowledged as a potential source of bias. Therefore, the PCA solution and derived factor structure require confirmation in larger and independent cohorts to establish their robustness and generalizability.

In conclusion, the 1‐MET value observed in this sample of community‐dwelling older adults was lower than the standard value commonly reported in the literature. Body size, body composition, and cardiorespiratory fitness were all associated with resting VO_2_; however, PCA indicated that the variance of 1‐MET was more closely aligned with VO_2_peak than with markers of body size and composition. These findings support a potential role of cardiorespiratory fitness in the regulation of resting metabolic rate among older adults. Nevertheless, because the study was conducted in a predominantly female volunteer sample recruited from a single geographic area, the findings should be interpreted with caution and may not be directly generalizable to broader older populations. Further studies including larger, geographically diverse, and sex‐balanced cohorts are needed to confirm these observations.

## Author Contributions

Conceptualization, Gabriela Venturini, Paulo Farinatti, Felipe A. Cunha, and Nádia S. L. Silva; methodology, Gabriela Venturini, Carlos A. A. Ribeiro, Paulo Farinatti, and Nádia S. L. Silva; formal analysis, Paulo Farinatti and Felipe A. Cunha; investigation, Gabriela Venturini, Carlos A. A. Ribeiro, and Nádia S. L. Silva; resources, Paulo Farinatti and Felipe A. Cunha; data curation, Felipe A. Cunha and Nádia S. L. Silva; writing–original draft preparation, Gabriela Venturini, Felipe A. Cunha, and Nádia S. L. Silva; writing–review and editing, Paulo Farinatti and Nádia S. L. Silva.; supervision, Felipe A. Cunha and Nádia S. L. Silva; project administration, Nádia S. L. Silva; funding acquisition, Paulo Farinatti and Felipe A. Cunha.

## Funding

This research was funded by a grant from the Carlos Chagas Filho Foundation for Research Support in the State of Rio de Janeiro (FAPERJ, E‐26/202.880/2017, recipient Paulo Farinatti; E‐26/200.514/2018, recipient Gabriela Venturini), National Council for Technological and Scientific Development (CNPq, 303629/2019‐3, recipient Paulo Farinatti; 102166/2017‐0 recipient Carlos A. A. Ribeiro), and Coordination for the Improvement of Higher Education Personnel (CAPES, Finance Code 001).

## Disclosure

All authors have read and agreed to the published version of the manuscript.

## Conflicts of Interest

The authors declare no conflicts of interest.

## Data Availability

The data that support the findings of this study are available on request from the corresponding author. Data are not publicly available because they contain potentially identifiable clinical information, and public deposition was not covered by the participants’ informed consent or the ethics committee approval.
